# The Dynamic Interest in Topics within the Biomedical Scientific Community

**DOI:** 10.1371/journal.pone.0006544

**Published:** 2009-08-07

**Authors:** Frederic Michon, Mark Tummers

**Affiliations:** Institute of Biotechnology, Developmental Biology Program, University of Helsinki, Helsinki, Finland; Harvard University, United States of America

## Abstract

The increase in the size of the scientific community created an explosion in scientific production. We have analyzed the dynamics of biomedical scientific output during 1957–2007 by applying a bibliometric analysis of the PubMed database using different keywords representing specific biomedical topics. With the assumption that increased scientific interest will result in increased scientific output, we compared the output of specific topics to that of all scientific output. This analysis resulted in three broad categories of topics; those that follow the general trend of all scientific output, those that show highly variable output, and attractive topics which are new and grow explosively. The analysis of the citation impact of the scientific output resulted in a typical longtail distribution: the majority of journals and articles are of very low impact. This distribution has remained unchanged since 1957, although the interests of scientists must have shifted in this period. We therefore analyzed the distribution of articles in top journals and lower impact journals over time for the attractive topics. Novelty is rewarded by publication in top journals. Over time more articles are published in low impact journals progressively creating the longtail distribution, signifying acceptance of the topic by the community. There can be a gap of years between novelty and acceptance. Within topics temporary novelty is created with new subtopics. In conclusion, the longtail distribution is the foundation of the scientific output of the scientific community and can be used to examine different aspects of science practice.

## Introduction

Communication is essential to the practice of science. Modern scientists have several communication channels at their disposal for the interaction with the scientific community; the most important one is the publication in the peer-reviewed scientific journal [1]. Peer review is the practice where a manuscript is scrutinized for its potential for publication by the journal editor, and then sent out to a selected group of peers: fellow scientists with expertise knowledge in the area of the manuscript. The peers review the quality of the manuscript and based on their reviews the manuscript is rejected or accepted, often with modifications. It is generally seen as the best system available to guarantee that the work is conforming to the standards of scientific practice. Most biological and biomedical journals use the peer review system.

In the last 50 years the biomedical scientific community has dramatically increased in size, although exact data on this topic is incomplete and intermittent. One way to measure growth of science is by looking at the number of scientists. The United States Department of Labor publishes every two years the Occupational Outlook Handbook which contains the employment figures for various occupations and it shows that the number of biological scientists have increased with 50% from 1994 to 2006 [2]–[3]. Membership of a scientific society can be used as an indicator of the size of the scientific community, as well as the membership of specific scientific research institutes, and these have been growing on a semi-logarithmic scale [4]. The changes in the scientific community over the last 50 years have indubitably had an effect on the practice of scientific communication and affected the dynamics of scientific production or output. For the sake of convenience we defined biomedical scientific output in this paper as all the original publications and reviews that were present in the PubMed database [5]. This database is not a complete record, but it is one of the most comprehensive search engines for biological and biomedical research.

There are several tools available that attempt to analyze qualitative aspects of scientific publication, of which the best known is the Impact Factor (IF) maintained by Thomson Scientific, which measures the citation impact of journals [6]–[7]. The IF is calculated based on how many times articles from a journal have been cited in the past two years. More citations should theoretically indicate higher impact on the scientific community, although there is a longstanding and passionate debate taking place on the merits of the IF as a measurement of quality [8]–[10]. An alternative to the IF is the SCImago journal rank indicator (SJR) which also ranks scientific journals based on citation data [11]. The advantages of the SJR are that it is an open-access source, covers a wider range of journals and uses weighted citations [12]. An alternative to ranking journals is the ranking of individual scientists. The most commonly used tool for this purpose is the H-index [13] which is also using citation data.

The bibliometric analysis examines the output of science [14]. The bibliometric study can show changes in the interest of a scientific community over time. In this study we make a generalized assumption: we equal the scientific community to their scientific output, and we equal the topics on which the scientists publish to the interests of the scientific community. We investigated the dynamics of the biomedical scientific output via the peer-reviewed journals over the period 1957–2007 by analysis of the PubMed records [5] and the SCImago database [11], documenting the great increase in scientific production; the dynamics of scientific production for various topics as indicated by a bibliometric analysis of biomedical key words; the sudden appearance of the review article as a means of scientific publication; the concomitant creation of more and more specialized journals supporting the mass of scientific production; the longtail distribution of the citation impact of journals and articles; the meaning of the longtail distribution, and we show how the longtail distribution is generated over time by examining attractive topics.

## Results

### The Longtail distribution in the citation impact of scientific journals

**Figure 1:**
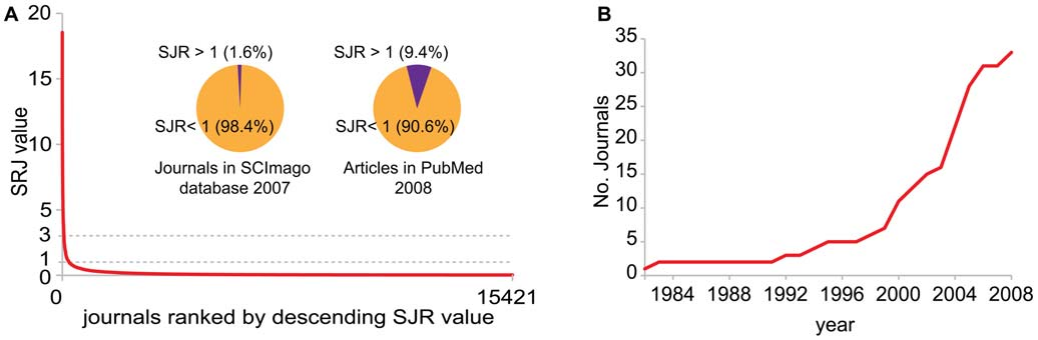
The longtail of SJR distribution among scientific publications. A, when journals are hierarchically organized according to their SJR value the longtail distribution becomes apparent (source: SCImago database). There are relatively only a few high impact factor/SJR journals (1.6% has an SJR value of above 1). The vast majority of scientific journals have a low SJR resulting in a typical longtail distribution. B, Expansion of journals in the Nature Publishing Group (NPG) with the word “Nature” in the title. In 1982 there is still only one journal in the NPG group: Nature. In 1999 the NPG has expanded to 7 titles, and in 2008 to 33 titles.

We used the SCImago journal rank indicator (SJR) [11] as an alternative to the Impact Factor (IF) because it is an open access source. Both measure the impact of a journal based on citations of articles in these journals. Among the large amount of journals ranked by SCImago, we have selected those which have published at least 1 citable document during the last 3 years (15,421 journals in 2007). The SJR values ranged from 18.542 to 0.032 with a highly specific distribution, the longtail distribution, originally described by the social economist Vilfredo Pareto [15] (Fig. 1A). High and medium impact journals are in the minority. The majority of journals have a minimal citation impact. Of the 15,421 journals 14,756 have a SJR value of lower than 0.5. There are of 427 journals with a SJR value between 0.5 and 1.0, 238 journals with a SJR>1, and only 59 journals with a SJR>3, the top journals (the journal Development has a SJR of 3 in 2007). The first pie chart in figure 1A summarizes this distribution, showing that only 1.6% of all journals have an SJR>1. The second pie chart shows the actual amount articles in these journals (using the PubMed database as the source). Interestingly, the top 1.6% of all journals produces about 9.4% of all articles. In conclusion, the distribution of scientific journals according to their impact creates a longtail distribution: only a small percentage has high impact, most journals are of very low impact, and it is this bulk of low impact journals that creates the longtail.

Are so few top journals because rarely new ones are created? This doesn't seem to be quite true. A common phenomenon is the creation new titles under an existing umbrella or journal brand. For instance, the first issue of Nature dates back to 1869, and more recently the Nature Publishing Group (NPG) has expanded the highly recognizable name of Nature into a series of similarly named journals such as Nature Genetics, Nature Medicine etc, increasing the Nature series to 5 journals in 1997, and 33 in 2008 (Fig. 1B). The NPG has even more high impact journals under its wings and according to their website it will publish no less than 81 scientific journals in 2009. Similarly BioMed Central has rapidly increased the number of journal titles by creating new titles and assimilating existing ones. Another well known example of the expansion of a series of journal titles within one publishing entity is the PLoS organization currently with 7 titles. This phenomenon is not entirely new. The Trend series started in 1976 with Trends in Biochemical sciences and saw most of its expansion to new titles in the 80s and 90s. High impact journals are therefore constantly created.

### Global increase of the scientific production

**Figure 2:**
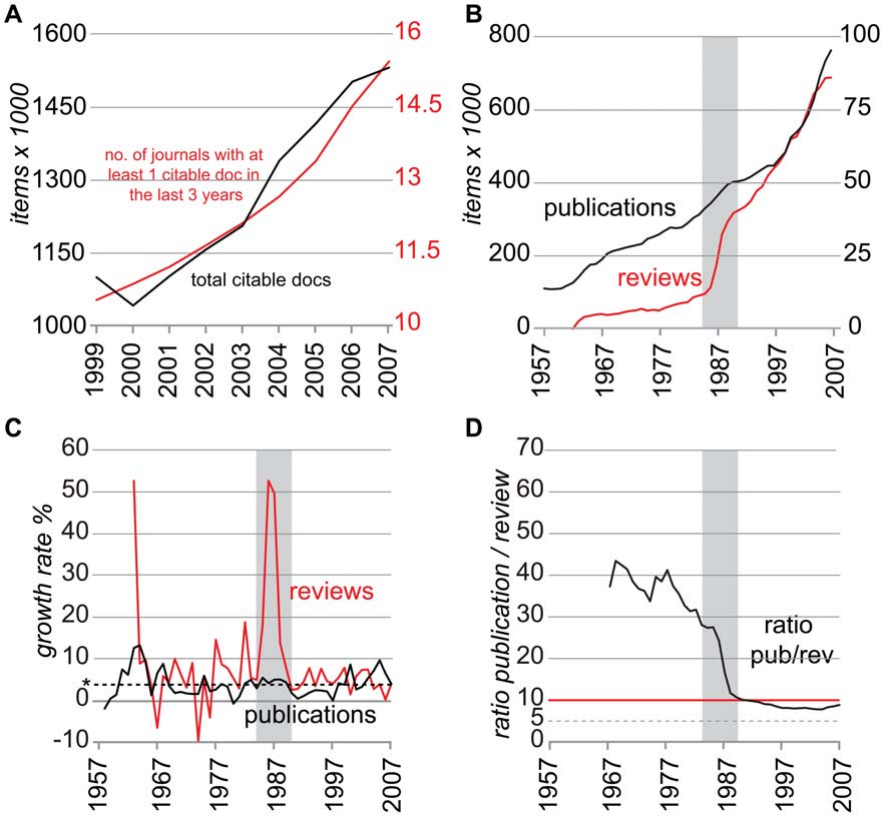
The dynamic increase in scientific output. A, the increase in journals for the period 1999–2007 matches that of the increase in publications (source: SCImago database). B, The total scientific output has increased from 110,568 to 763,041 documents in the period 1957 to 2007 and small fluctuations can be seen in total scientific production (source: PubMed). In contrast the output of reviews shows three distinct phases. C, The annual growth rates of scientific output show that the total growth rate shows distinct periods of growth rates above and below average (line denoted by asterisk shows average growth rate). The growth rate of annual scientific output is mostly positive explaining the constant increase in scientific production over the period of 50 years. Interestingly the growth rate of the reviews indexed in the PubMed database shows a large peak during the years 1986–1990, explaining the sudden increase in review output in the same period (B–C). This dynamic change becomes also apparent during changes in the annual ratio of publications per review (D) where a drastic shift can be seen when the ratio drops from over 30 publications per review during the early 80s to below 10 after 1990.

The SCImago database shows that the increase in scientific journals has kept up to date with the production of scientific articles, having a constant ratio of roughly 1 in 100 (Fig. 2A). Currently more than 34,000 peer-reviewed journals are present in the PubMed database, and over 5,000 of these journals are fully indexed. It is probably the most complete database for biomedical and biological research. To check the completeness of the PubMed record we randomly picked 20 journals with a SJR>1 and compared the PubMed record with the content of the electronic archive of the journal at an interval of 10 years. Of two journals the electronic record was unavailable. The PubMed records of 13 journals were fully indexed, of 4 the record was mostly complete, and 1 was incomplete (Table S1). From 1957 to 2007 there has been a tremendous increase in scientific output recorded in the PubMed database (Fig. 2B) going from a bit over 110,000 publications in the year 1957 to over 760,000 publications in the year 2007. The increase in scientific output has not been steady over the years and small fluctuations can be seen in the yearly growth rate (Fig. 2C).

This increase in scientific output from 1957 to 2007 involves not only publications that present original research, but also review articles. It is of importance to realize that PubMed uses internal criteria for labeling a publication as a review, where the main criterion is that a review publication has the intention of summarizing or reviewing existing publications. The definition of review should be therefore largely independent of historic definitions, which allows for an analysis of the prevalence of reviews versus all publications in the PubMed database. In 1958, the very first review appears according to the standards set by the PubMed database, while in 2007 PubMed has referenced no less than 86,110 reviews. The growth rate of the reviews published each year as indexed by the PubMed database has not been stable over the years. While the increase in total publications shows more or less a steady curve over the last 50 years, the dynamics of review publication shows three distinct phases (Fig. 2B–D). The period 1957–1985 shows a steady growth in review output, with an initial growth spurt in the very beginning.

For the period 1981–1985 there were 1.4 times more reviews than in 1976–1980. However, for the period 1986–1990 there were 2.7 times more reviews than in 1981–1985. For the following period of 5 years there were 1.5 times more reviews. There is therefore a remarkable peak in the growth rate of review output during 1986–1990 (Fig. 2C). The increase of reviews has led to a radical change in the ratio of publications per review (Fig. 2D). The ratio publication per review dropped from 40 original publications per review during the 70s to less than 10 in 1991 and stayed below 10 ever since. The question is whether this means that the review concept became an integral part of the common scientific practice in this short period of time. We could not entirely pinpoint the exact origin of this increase in review output for the period of 1986–1990. We tested some keywords and noticed that the medical orientated review seemed to constitute a large percentage of all reviews. Keywords such as medical gave in the critical period (1986–1990) a 13.4 times increase in the amount of reviews (compared to 2.7 times increase for all reviews). Or in other terms, the keyword medical constituted 2.9% of all reviews in the period 1981–1985, while in the period 1986–1990 the percentage of medical reviews grew to 17% of all reviews. A similar trend was seen for other medical keywords (clinical, 3.7 times increase; care, 4.0 times increase; patient, 4.5 times increase; case report, 7.3 times increase). This didn't mean that there wasn't a dramatic increase in the amount of reviews on non-medical topics, but we couldn't pinpoint one that was giving a major quantitative contribution to all reviews. For instance, the keywords developmental biology gave an 8.9 times increase, but at the same time it only constituted 0.2% of all reviews in this critical period. Similarly fibroblast growth factor gave a 6.5 times increase but this was only 0.1% of all reviews. A more thorough analysis of this critical period where the review article became predominant would certainly be interesting.

### The dynamics in scientific output for various keywords

To have a more detailed overview of the scientific output dynamic, we analyzed a set of keywords representing different biomedical topics in the PubMed database for the years 1957–2007. We initially selected a mix of keywords focused on biomedical sciences, corresponding to old and new disciplines, narrow and broad topics (cancer, stem cell, cell cycle, evolution, mollusc, cholera, retinoic acid (RA), Lysergic Acid Diethylamide (LSD), recombinant DNA (rDNA), Human Immunodeficiency Virus (HIV), Wnt, Bovine Spongiform Encephalopathy (BSE), apoptosis, microRNA (miRNA), Fibroblast Growth Factor (FGF) and Ectodysplasin (Eda)).

**Figure 3:**
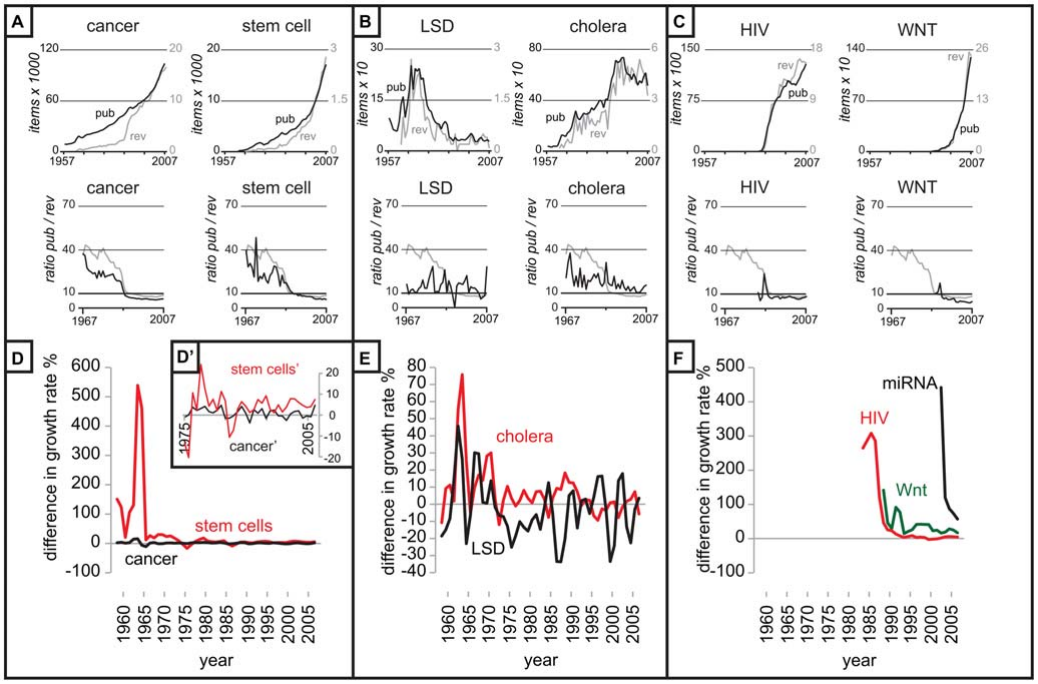
The analysis of the dynamics in scientific output for a selection of keywords representing different biomedical topics. In panel A, B and C the output in scientific publications and reviews, and the ratio of publications per review is depicted (source: PubMed). Three broad categories can be discriminated. Panel A shows the typical keywords cancer and stem cell which resemble the pattern of total scientific output (Fig. 2B), B shows the atypical keywords LSD and cholera which show highly variable patterns, and C shows the attractive topics HIV and Wnt which show rapid growth and a low publication per review ratio. The grey line in the publication review ratio figures represents the ratio for all scientific output (Fig. 2D). In D, E and F the topical growth rates (%) of the keywords subtracted by the growth rate of all scientific output is shown (averaged over a period of 2 years). A value of 0 therefore means that the growth rate of the topic is exactly the same as that of all scientific output. General topics as cancer remain close to the zero line, atypical topics such as cholera and LSD show highly variable pattens of mixed negative and positive growth rates. Attractive topics such as HIV, miRNA and Wnt start of with extremely high difference in growth rates which diminish over time. Insert D' shows that the variation within the topic of stem cell is greater than for cancer.

Analysis of the keywords showed that three broad categories could be distinguished based on their output pattern (Fig. 3). There are typical patterns that follow the general trend of all scientific output. There are atypical keywords that show highly variable patterns. The atypical patterns are so variable that there is no one definition possible for them except that large variations in the scientific output exist, compared to all scientific output. And finally there is a category of keywords that represent new topics and show a very fast increase of scientific output compared to the increase in the output of all items in the PubMed database, which we labeled as attractive topics.

The keywords cancer, stem cell, cell cycle and evolution approximated the typical pattern of all scientific output. These topics represent vast bodies of work and are sometimes very general in nature, such as cancer and evolution. In January 2009, the query cancer in PubMed resulted in 2,074,887 return items representing scientific publications for the period 1957–2007. For the year 2007 alone 103,842 hits are returned in the PubMed database (corresponding to over 300 publications per day) on a total of 763,041 publications referenced by PubMed in that year (Fig. 3A). A general topic such as cancer therefore has an overwhelming amount of scientific output each and every day. And this output is constantly increasing. The topic of stem cells (Fig. 3A) is slightly smaller, yet there is still a substantial output, as is also the case for cell cycle and evolution (Dataset S1). These topics exist mostly from the beginning (taking 1957 as starting point) and have been accepted topics within the community. Although the difference in growth rate compared to that of all scientific output for the keyword cancer remains close to the neutral line of 0%, showing minimal variation in the growth rate, for the topic of stem cells there appears to have been several phases with increased interest, and each phase resulting in an increase in the difference in growth rates (Fig. 3D–D'). The ratio of publication per review of typical topics shows usually the distinctive dip in this ratio during the 80s (Fig. 3A). In conclusion general topics therefore appear to follow the overall trend in scientific production, although this is not always the case for general topics.

The keyword mollusc also entails a broad range of research interests but shows a highly variable pattern in scientific output, and is an example of atypical topics. This is a characteristic shared also by other keywords we investigated such as cholera, retinoic acid, LSD and recombinant DNA (Fig. 3B, Dataset S1). Of some of these keywords the history is quite well known, such as LSD, a field of research which had a main focus of interest in the 60s. This is confirmed by the PubMed data which shows a steady decline in output since the early 70s representing the reduced interest in the community (Fig. 3B). The topic cholera has seen a steady growth in scientific output till the early 90s when the interest in the topic seems to have disappeared, and scientific output starts to decrease. It is nowadays labeled as one of the neglected diseases [16], signifying amongst other things a lack of attention to the disease in the media and research budgets. LSD is showing mostly negative growth rates after the initial popular phase, and the interest in cholera has mostly decreased since the 1990s (Fig. 3E). The analysis in scientific output based on keyword analysis of the PubMed database therefore corresponds to known historic trends in scientific interest. It is therefore very well possible that scientific output is directly correlated to scientific interest.

The final dynamic profile of scientific output is on relatively new subjects such HIV, BSE, apoptosis, Wnt, miRNA, Fibroblast Growth Factor and Ectodysplasin. They could be seen as the attractive topics in science (Fig. 3C, dataset S1). All of these subjects exhibit a fast increase of the scientific output with growth rates of well above average (Fig. 3F). The average growth rate of the total scientific output is 4%, while that of HIV is 60%, Wnt is 44%, BSE is 36%, and apoptosis is 41%. The steep increase in scientific output is tentatively caused by an increase in the amount of interest in the scientific community. Interestingly, the ratio publication per review of the attractive topics is mostly under the average of 8.9 already from the onset of the appearance of these topics in the PubMed database (Fig. 3C), and most of them are close to, or under the publication per review ratio of 5, well below average. This indicates a high interest from the active members and interested parties in these new subjects, and a need to summarize the rapid developments on the new subject. The difference in growth rates compared to all scientific output starts off extremely high and slowly levels off (Fig. 3F). Therefore, it is fairly easy to distinguish a popular or attractive topic since they exhibit a higher than normal growth rate in scientific production, and more than an average amount of reviews are produced.

The classification based on the handpicked keywords was validated using a comprehensive list of keywords based on the subjects of the Gordon Research Conference meetings in the year 2000 [17], generating 67 extra patterns on scientific output, and an additional 8 topics concerning signaling pathway were selected based on two review papers (Dataset S2) [18]–[19]. The patterns could be matched to the three categories or typical, atypical and attractive topics, which became apparent with the analysis of the handpicked keywords, indicating that the categorization was robust.

The difference in growth rates, that is the growth rate of a topic subtracted with that of all scientific output in the PubMed database, gives possibly a better view on the interest of the scientific community (Fig. 3D–F). Cancer shows almost identical growth rates compared to all scientific output, explaining why its contribution to scientific output never really has changed much, but the keyword stem cell shows several phases above the neutral line of 0% indicating several phases of increased scientific interest over time. The difference in growth rates of attractive topics are all well above the neutral line of 0% (Fig. 3F). Comparing the growth rates of a scientific topic with that of all scientific publication therefore should give a decent indication of the interest of the scientific community for a topic, especially when it is combined with an analysis of the publication per review ratio.

### Scientific novelty and acceptance by the scientific community

**Figure 4:**
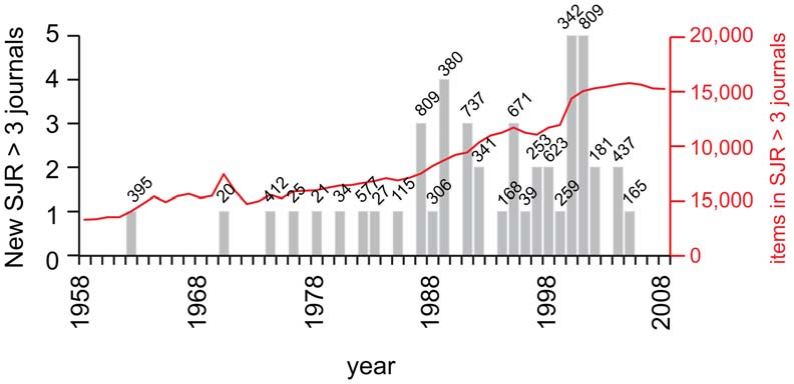
The introduction of journals with a SRJ>3 in the year 2007, from 1958 to 2008. In 1957 there are already 12 journal titles present which published in this particular year 2,569 articles (source: PubMed). Over the years new journals are intermittently introduced (figure on top of the bar represent the average articles per year for the journal titles introduced that year). The red line represents the actual amount of articles in SJR>3 journals retrieved from PubMed.

**Figure 5:**
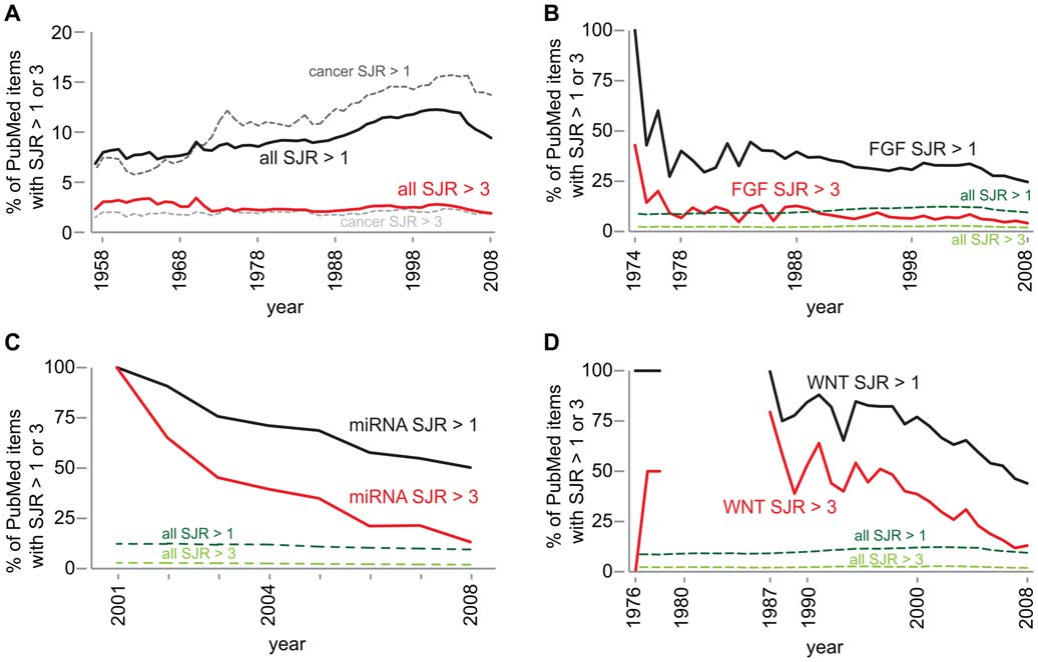
The historic trends in the longtail distribution of articles in the PubMed database according to their citation impact. A, two SJR values (>3 and>1) were taken to represent the longtail distribution as can be seen in Figure 1A and examined over time. Interestingly, the percentage of articles in SJR>3 journals (top 59 journals) remains steady over time (2.5% of total scientific output), where the articles in SJR>1 journals (top 238 journals) show more variation. The values for the articles on cancer are very similar to that of all scientific output (dashed lines). B, for novel topics, such as FGF, the distribution is rather different. The first articles are mainly published in the top journals. Over time the distribution shifts towards that of all scientific output (dashed lines). C, the same can be seen for a recent attractive topic, miRNA, where initially all articles were in the top 59 journals. Slowly the distribution shifts towards lower impact journals. D, the topic of Wnt shows another interesting phenomenon. The topic was originally introduced in 1976, and publication in top journals followed for the next two years. This is followed by a gap of almost a decade before the topic is re-introduced, once again in top journals, and once again there is the typical progressive change towards the typical longtail distribution when the scientific output on the topic increases.

To further investigate the biomedical scientific output we examined the distribution of the citation impact of the items in the PubMed database. Figure 1 shows the static picture of the longtail distribution of the citation impact of the items in the PubMed database. We used two values, that of journals with a SJR>3 (top 59 journals) and a SJR>1 (top 238 journals), to examine the longtail distribution over time. This analysis is not entirely accurate since we took the SJR values of 2007 and went back to the year 1957. Naturally, the citation impact of some journals has shifted over time, some journals did not always exist, and of course the concept of an impact factor didn't always exist [7]. If we look at the history of the top 59 journals than we see that in 1957 there are already 12 journal titles which are themselves responsible for 2,569 articles on a total of 110,593 in that year, which equals 2.3%. Over the years intermittently new journal titles are added in the contemporary SRJ>3 category (Fig. 4). It results in a very stable picture for the amount of articles in journals with an SJR>3, with a steady percentage of around 2.5% of all articles found in journals with a SJR>3 (Fig. 5A). More variation can be found for the items in journals with an SJR>1. The percentage has gone from 7.5%, past 12%, and is currently at 9.4%. Therefore about 1 in 10 of all articles found in PubMed is published in the top 238 journals according to the SJR ranking. That still leaves the other 9 articles in journals that constitute the longtail. A general topic such as cancer that has been around since the beginning shows a similar distribution with on average 1.9% of all PubMed items in journals with SJR>3 (Fig. 5A). There appears to be more midrange publications on the topic of cancer since the values of items in journals with SJR>1 are mostly larger than for all scientific output, but the difference is minimal.

The difference between the historic SJR distributions of all scientific output and cancer which remain mostly stable (Fig. 5A), and that of newly introduced topics such as FGF, miRNA and Wnt is remarkable (Fig. 5B–D). The point of introduction of a new topic usually constitutes a handful of publications. Novelty is indeed rewarded since these items end up mostly in SJR>1 or even SJR>3 journals (This is valid for the topics we investigated; we claim by no means that this is true for all cases). FGF was introduced by no less than 7 papers in the year 1974 of which 3 were published in SJR>3 journals, and all were in SJR>1 journals. This is followed by a rapid increase in scientific output on the topic, where it is interesting to see that relatively more and more papers are published outside the top journals each year. The longtail distribution is absent at the onset of a novel topic, and with an continual increase in scientific output the longtail distribution is generated and strengthened, causing the distribution slowly to shift towards the typical longtail distribution of all scientific output. This could be seen as a mark of acceptance of a topic within the scientific community as a valid and productive research topic.

A similar pattern can be observed for the topic of miRNA. The year of introduction features 5 articles and all of them are in SJR>3 journals (Fig. 5C). Also here there is a rapid increase in scientific output over the following years which is characterized by a relative and progressive increase in publications outside the SJR>1 journals.

The keyword analysis of Wnt (Fig. 5D) shows another interesting feature which also exists in the pattern for Ectodysplasin (Dataset S1). The first five publications in the year 1976–1978 are published in high impact journals, followed by a long gap with no output on the topic. Novelty was in this case awarded since the original publications ended up in excellent journals, but the scientific community didn't follow up immediately on these publications. In the PubMed record there is a gap till the year 1987. In order to check whether this gap is real or an incompleteness of the PubMed record we analyzed the references of the papers on Wnt and Wingless in 1987. These papers should in theory cite the previous work on the topic.

This analysis showed that the gap could be shortened to some degree (1979–1982) since the vertebrate form of the Wingless gene was known as *Int-1* and publication on this gene started in 1982 in a line of research unrelated to Wingless (Table S2). The *Int-1* gene was identified as a frequent target of insertional activation by the mouse mammary tumor virus (MMTV) [20]. Only in 1990 papers started referring to *Int* as *Wnt*. In 1991 this nomenclature became official [21]. The two research lines on *Int-1* (or *Wnt*) and *Wingless* didn't become connected till 1987 when it was discovered that they were the same gene in different organisms [22]. The reference analysis also showed that two references on Wingless are missing in the PubMed database. One is a PhD thesis on the *Wingless* gene in *Drosophila* from 1985 [23], and one is a research article on *Wingless* in *Drosophila* from 1986 [24]. However, after this transition phase where the Int-1 and Wingless research lines were merged, there is once again a relative and progressive increase in the publication profile towards journals with an SJR<1. Also for the keywords Wnt and wingless the longtail distribution for the citation impact is progressively strengthened.

### Novelty and longtail distribution in subtopics

**Figure 6:**
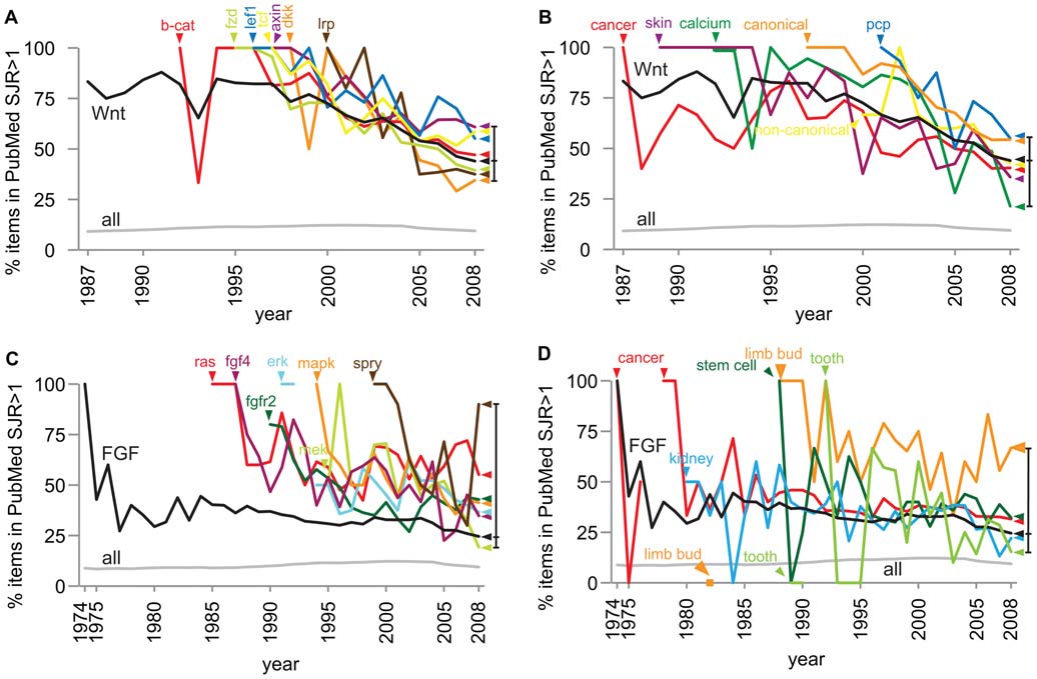
The historic analysis of the longtail distribution of articles on various topics in the PubMed database in journals with a SJR>1 within a single topic. A, various components of the Wnt pathway, beta-catenin (b-cat), frizzled (fzd), lymphoid enhancer binding factor 1 (Lef1), axin, Dickkopf (dkk), and Low density lipoprotein receptor-related protein (Lrp), are introduced at different time points, and this particular selection of keywords are on average all introduced in journals with an SJR value higher than that for all articles on Wnt. In 2008 the subtopics are distributed around the reference value of all Wnt publications. B, a similar situation was found in a topical analysis; cancer, skin, calcium, canonical, non-canonical, planar cell polarity (pcp). Most subtopics of the Wnt topic mostly start higher than the Wnt reference line, but not always. C, the components of FGF signaling, Rat sarcoma (Ras), fibroblast growth factor 4 (Fgf4), fibroblast growth factor receptor 2 (Fgfr2), Mitogen-activated protein kinase (Erk, Mapk and Mek), and Sprouty (spry), are also introduced in high impact journals compared to the rest of the publications on this topic. D, the subtopics on FGF signaling, cancer, kidney, limb bud, stem cell, and tooth, also often start in higher than usual impact journals, and the patterns are more variable due to the small amount of publications on many of these subtopics.

In order to investigate the novelty concept further we examined individual components of the Wnt and FGF signaling pathways, and other subtopics of these pathways. When taking all articles on the topic of Wnt published in a journal with an SJR>1 as a reference we can see that the introduction of individual novel components of the Wnt pathway is usually rewarded (Fig. 6A). All subtopics start above the reference line, signifying that an article containing the novelty of a new component of the Wnt pathway will on average end up in a higher impact journal than just any article on Wnt. Over time the subtopics approach or dip below the reference line, and when taking 2008 as a static reference all the subtopics are distributed below and above the reference of all Wnt publications with SJR>1. A similar distribution was found for SJR>3 (data not shown). In conclusion, the average of all articles on the novel component of the main topic are introduced on average with an SJR value higher that of all Wnt articles, and after some years the average impact of the articles of the subtopic decreases, and ends up above and below the average impact of all Wnt articles.

A similar picture emerges from the topical analysis of Wnt signaling (Fig. 6B). Most topics start above the reference line of all Wnt publications, although not all. However, once again, the subtopics are distributed in 2008 below and above the reference line of all Wnt publications. It has to be noted though that not all sub topics are created equally. The topic of planar cell polarity combined with Wnt signaling has managed to stay above the reference line since the introduction, indicating that relatively more high impact publications are published on this topic than for other Wnt topics investigated here.

A similar picture emerges for the FGF signaling pathway (Fig. 6C–D). Novel components are rewarded with an initial high impact profile, which after time moves towards the reference distribution of all FGF articles. The picture is slightly more chaotic for the topical components of FGF signaling. In general subtopics of FGF start with a high SJR profile and move over time to the reference line. Some smaller subtopics show more variation due to a lesser amount of publications. In conclusion, introducing novelty to an existing subject is also often awarded with a publication, on average, in a higher impact journal than for all articles on this topic.

### Tactical use of keywords

In principle keywords can be used in a tactical manner by authors to increase the visibility of their paper. To test whether this is the case we analyzed 100 random abstracts of publications in the PubMed database for the keywords cancer and Wnt, and 50 random abstracts for the combination of the keywords Wnt and cancer. For the keyword cancer 93% of the abstracts were on topic, 5% were not, and 2% could not be determined. The keyword cancer is therefore hardly used as a buzzword. The situation was slightly different for Wnt, where only 79% was on topic, 19% was on unrelated topics, and 2% could not be determined. The combination of both keywords Wnt and cancer gave an intermediary result, with 86% of all abstracts on topic, and 10% of the abstracts just on the topic of Wnt. Not a single abstract was just on cancer, and 4% could not be determined. The greater incidence of unrelated usage of the keyword Wnt could indicate that it is perceived as a topic with a high profile.

## Discussion

### Overwhelming scientific production?

The obvious message of this paper is that there is an overwhelming increase in scientific production for almost every topic conceivable in the biomedical field. Even for subjects that result in a modest scientific output and are experiencing a diminishing interest, such as BSE, the interested scientist is still faced with 13 papers in the month January of 2009. The subject miRNA is one of the latest “attractive” topics, and in January 2009 there were 260 papers on miRNA and 36 reviews. It is physically impossible to keep a perfect overview on general topics such as cancer where for the same month you would have to read 11,215 new papers, or 1,220 review articles.

The point has been made in the past that the upkeep of reviewing scientific literature is manageable because there is only a small core of significant literature which is presented mostly in the corresponding core of high impact journals [25]. This conclusion was based on an old database (Science Citation Index CD-ROM). The old database showed that in 1996 the top 100 journals account for 44% of the cited articles. The current situation is different. The top 59 journals in the year 2008 (according to the SJR ranking) accounted for 1.9% of all publications in 2008. If we expand our selection of journals to the top 238, we still only have covered 9.4% of all items in the PubMed database. In the 1996 database 2,000 journals accounted for 95% of cited articles. In 2007, according to the SCImago database, 2,000 journals account for 65% of the cited articles (and 2,000 journals equals 417,663 articles).

Low impact journals might be obscure for many of us, but most journals do receive citations. Only 6% of all 15,421 journals in the SCImago database for the year 2007 didn't manage to get a single citation over the past three years. The majority of journals are being cited, albeit minimally. What is striking is that the first 2,000 journals only account for 37.6% of all references. And therefore all the journals outside the top 2,000 journals (13,000+journals) actually provide 62.4% of all references. This means that the longtail of low impact journals actually is responsible for the majority of the references that drive the high impact of the core group of high impact journals. All scientific output, high and low impact alike, is linked together in the game of impact factor and prestige.

The scientific community has devised tools to deal with the problem of overwhelming information. Academic search engines such as PubMed allow the scientist to redefine a subject to narrower parameters limiting the information overflow. Another tool is the database, where people knowledgeable in the field collect already published results. The “Bite-it database” created in 1996 collects expression patterns during tooth development based on patterns in the literature [26]. The “Wnt Home page” created in 1999, presents all the key results related to Wnt signaling [27]. Attending conferences will help the scientist with getting updated on the latest research. However, still choices have to be made in reviewing the literature, and it is an interesting hypothesis that the concept of review article was institutionalized to facilitate scientists to have an overview of the most relevant literature regarding a specific topic. With increased literature you can expect a correlated increase in the need for an overview of the literature. However, our data suggests that this is not the case. Over a short period of 5 years (1986–1990) the ratio publication per review plummeted (Fig. 2B–D), meaning that the increase in the amount of reviews was more progressive than that of regular articles in this period. PubMed has internal criteria to determine whether an article is a review. This suggests that the rapid increase of review articles in this period is a real phenomenon. A small survey of different keywords for this period suggested that the increase in reviews was probably mostly driven by an increase in reviews by the medical community (case reports, clinical reviews etc). Another explanation for the sudden attractiveness of the review is that review article can be used as a means to raise the impact factor of publications and journals. Review articles tend to be overrepresented in highly cited papers [28]–[29]. Besides these speculations it is clear is that the publication per review ratio, especially in more contemporary times, is accurate enough to reliably determine the interest in a topic. Less publication per review tends to indicate more interest in the scientific community for the topic.

### The longtail of scientific publication

The distribution of scientific journals plotted according to their SJR value (citation impact) seen in figure 1 is a typical example of the longtail distribution first described by the social economist Pareto [15]. While the high impact journals are usually presented as the flagships of scientific publication, the majority of scientific output is published in low impact journals. In the movie industry Pareto's principle is known as the 20–80 rule; 20% of the released movies are hits, and 80% are not [30]. A common mistake is to think that only the 20% hit movies make money. The non-hit movies usually make some money as well, but much less. This smaller profit margin is compensated by the larger amount of them: the longtail. We can apply the longtail effect also in scientific publication, and it would be interesting to see the profit margins on low and high impact journals. In the science publishing industry 9.4% of the work is published on a journal with a high SJR value (SJR>1) and 90.6% is not. There are a lot more of the low impact journals which could render them more profitable despite lower profit margins. In other industries the internet facilitated the marketing of the components of the longtail. Is a similar phenomenon present in science publishing and has the internet created an extended market for low impact science?

### Interest, novelty and acceptance

Novelty is rewarded. We have seen that novel topics are often introduced in the top journals (Fig. 4–5), although this might not always be the case. The decision to publish here is possibly mostly determined by the editorial component of the journal involved and not by the scientific community as such, although of course the peer reviewers might equally facilitate and inhibit the publication of certain topics. When a novel topic is introduced to the scientific community there is no longtail distribution of the citation impact. Often all initial articles are published in high impact journals. Over time more and more articles are published on the topic and there is a shift in impact distribution. Relatively more and more articles will be published in journals with lower citation impact. Over time the distribution will approach the impact distribution of all scientific publications. The shift towards lower impact and the increase in scientific output can be seen the acceptance of a topic by the scientific community, that is, the topic is embraced by all aspects of the scientific community, top research labs, the midfield, and the fringe alike.

Acceptance does not always occur. Topics that are initially introduced in high impact journals can be followed by a period of silence. The topic of Wnt or Wingless was first introduced by the description of Wingless mutants in 1976 [31]. Publication on this topic was sporadic till 1987. In this year the topic was merged with the research on the oncogene *Int-1* when it was discovered that the drosophila *wingless* gene was homolog of *Int-1* [22], [32]. A few years later the *int-1* gene was renamed *Wnt* [21]. Within a topic novelty is constantly generated and often this is rewarded again with the publication in journals with a higher impact than the research on the entire topic (Fig. 5B–D). After a while, the frequency of publication goes up and relatively more and more articles appear in lower impact journals. Some subtopics do tend to be rewarded higher than others so it might not be inadvisable to do an analysis if citation impact is important for the research, or researcher in question.

In short, the longtail distribution forms an integral part of the scientific community, that is, when we are allowed to define the community by its scientific output. The majority of scientific journals are of a low impact, but the mere number of them gives them a measurable impact on the high impact journals. They provide the references necessary for the high impact of the top journals. The scientists publishing in low impact journals validate novelty in top journals by publishing on the very same topics, i.e. scientific interest can be identified by the changes in the impact distribution towards the longtail pattern, especially when combined with an analysis of growth of scientific output, and examining the publication per review ratio.

## Methods

### Abbreviation list

IF, Impact Factor; SJR, SCImago Journal Rank indicator; NPG, Nature Publishing Group; RA, retinoic acid; LSD, Lysergic Acid Diethylamide; rDNA, Recombinant DNA; HIV, Human Immunodeficiency virus; Wnt, mnemonic for wingless-type MMTV integration site; BSE, Bovine Spongiform Encephalopathy; miRNA, microRNA; FGF, fibroblast growth factor; Eda, Ectodysplasin; TCF, transcription factor; Lgr5, leucine rich repeat containing G protein coupled receptor 5; LRP, low density lipoprotein receptor-related protein; GSK3, glycogen synthase kinase 3; PCP, planar cell polarity; Fgf4, Fibroblast growth factor 4; mapk, mitogen-activated protein kinase, ERK, mitogen-activated protein kinase; Mek, mitogen-activated protein kinase; RAS, Rat sarcoma; fgfr2, fibroblast growth factor receptor 2; Bmp, bone morphogenetic protein; Lef1, lymphoid enhancer binding factor 1.

### PubMed search command line template

To retrieve the number of publications (original articles and reviews) referenced in PubMed (http://www.ncbi.nlm.nih.gov/sites/entrez), we have used the following command line template: “(cancer) AND (“2007/01/01”[Publication Date] : “2007/12/31”[Publication Date])”. This command line has given the number of articles and reviews published during the year 2007 related to cancer. The retrieved articles during the early period of novel topics, was examined carefully in order to eliminate false positives.

### SCImago database

The SCImago research group website [11] has developed a portal including journal and country scientific indicators from the information contained in the Scopus database. The data was downloaded in excel files and used for figures 1A, 2A and some data in the discussion.

### Difference in Growth rate

The growth rate represents the dynamic changes in scientific production. We defined Scientific Production (SP) as the total amount of retrieved publications from PubMed database for a specific year. The total SP for a year is called SPt and the SP for a year for a specific topic “x” is named SPx. The growth rate of total scientific production in the year “n” (GRt_n_), used in Figure 3, is:
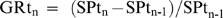



The growth rate for a specific topic in year “n” (GRx_n_) is therefore:
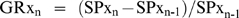



The difference in Growth rate is GRx_n_ - GRt_n_

Subsequently, the difference in growth rate of two years was averaged.

### SJR analysis of PubMed

The items in PubMed where analyzed with the following search command:

(“2007/01/01”[Publication Date] : “2007/12/31”[Publication Date]) AND ((“Cell”[Journal] OR “Science”[journal] … etc … AND (topic))

This command would give the results for the year 2007 regarding the topic in the journals specified. For SJR>3 the first 59 journals in the SJR ranking of 2007 were used. For SJR>1 the first 238 journals were used.

For analysis within a topic the last part of the search term was modified to:

((topic) AND (subtopic)))

Search terms used:

Cancer, Lysergic Acid diethylamide, stem cell, apoptosis, mollusc, recombinant DNA, retinoic acid, HIV, cholera, Bovine spongiform encephalopathy, cell cycle, evolution, bone morphogenetic protein, miRNA OR microRNA, ectodysplasin OR tabby, Wnt OR wingless (in combination with: frizzled, TCF, beta-catenin, axin, lgr5, disheveled, LRP, dickkopf, Lef1, GSK3, canonical, planar cell polarity, skin, non-canonical, calcium), Fibroblast growth factor (in combination with: fgf4 OR KS3 or hst or Fgfk or kFGF or Fgf-4 or hst-1 or Hstf-1, sprouty, fgf20, mapk, limb bud, ERK, stem cell, MEK, RAS, fgfr2, cancer, kidney, salivary gland).

## Acknowledgments

The authors would like to thank Irma Thesleff for her comments and support, and Pauliina Munne and Maria Jussila for the critical reading on the manuscript.

## Supporting Information

Table S1 completeness of the PubMed record tested on 20 random journals with SJR>1
(0.03 MB XLS)

Table S2 PubMed items on int-1
(0.03 MB XLS)

Dataset S1 Total and topical scientific output on selected topics
(0.58 MB XLS)

Dataset S2 Scientific output on topics derived from Gordon Research Conference meetings and additional signaling pathways
(0.35 MB XLS)
